# Validating the Methods to Process the Stress Path in Multiaxial High-Cycle Fatigue Criteria

**DOI:** 10.3390/ma14010206

**Published:** 2021-01-04

**Authors:** Jan Papuga, Eva Cízová, Aleksander Karolczuk

**Affiliations:** 1 Department of Instrumentation and Control Engineering, Faculty of Mechanical Engineering, Center of Advanced Aerospace Technology, Czech Technical University in Prague, Technická 4, 16607 Prague 6, Czech Republic; 2 Faculty of Mechanical Engineering, Czech Technical University in Prague, Technická 4, 16607 Prague 6, Czech Republic; eva.cizova@fs.cvut.cz; 3 Department of Mechanics and Machine Design, Opole University of Technology, ul. Mikołajczyka 5, 45-271 Opole, Poland

**Keywords:** multiaxial fatigue, shear stress path, minimum circumscribed circle method, minimum circumscribed ellipse method, maximum prismatic hull, moment of inertia method

## Abstract

The paper discusses one of the key features in the multiaxial fatigue strength evaluation—the procedure in which the stress path is analyzed to provide relevant measures of parameters required by multiaxial criteria. The selection of this procedure affects the complete equivalent stress derived for any multiaxial load combinations. Three major concepts—the minimum circumscribed circle, minimum circumscribed ellipse, and moment of inertia methods—are described. Analytical solutions of their evaluation for multiaxial stress state with components described by harmonic functions are provided. The concepts are validated on available experimental data when included into six different multiaxial fatigue strength criteria. The results show that the moment of inertia results in too conservative results. Differences between both methods of circumscribed entities are much smaller. There are indications however that the minimum circumscribed ellipse solution works better for critical plane criteria and for the criteria based on stress tensor transformation into the Ilyushin deviatoric space. On the other hand, the minimum circumscribed ellipse solution tends to shift integral criteria to the conservative side.

## 1. Introduction

Methods of multiaxial fatigue analysis should cope in some kind with one key problem: If the load history of individual stress tensor components is not proportional, the load path created by the end point of the stress vector gets multidimensional—it is not a straight line anymore. Most of practically used or developed fatigue estimation methods focus on detecting individual closed cycles in the load history, and on describing them by the most basic characteristics (mean value during the cycle, its amplitude). If the object of such description—the load path within the detected cycle—gets more complicated than the line is, the decision which geometric features could serve well for such a definition is not simple.

There are several types of multiaxial fatigue criteria, and the way the load path is treated differs for some of them (see e.g., [1,2]). The critical plane criteria evaluate the history of the stress tensor components on a specific critical plane. The projection of the load history onto a plane simplifies the calculation to some extent. The normal stress does not change its orientation. It is easily described by its mean value and by the amplitude. The description of the shear stress history is more problematic, because the load path projection onto the plane is not a linear object under non-proportional loading. The identical problem can be observed for integral multiaxial criteria, which integrate over all planes the output of the equivalent stress obtained from the calculation on each plane. Otherwise, the kind of processing the load path projection onto each of the evaluated planes by integral fatigue strength criteria is identical with the critical plane criteria. The third distinct group of multiaxial criteria works in the Ilyushin deviatoric space (see e.g., [3] or [1]). This is a 5D space into which the history of the six components of the stress tensor deviator can be transformed without any loss of information since the first invariant of the stress tensor deviator is zero and the stresses on the trace of the deviatoric tensor are linearly dependent. The load path thus should be described in all five dimensions.

One of the early attempts to summarize this problem, to describe it, and to propose the optimum solution can be found in the paper by Papadopoulos et al. [1]. In addition to validating the Papadopoulos criterion [4], it provides a complete analytical formulation for the problem of superposed axial and torsion harmonic loads as regards their projection onto evaluated planes or into the Ilyushin deviatoric space. These analytical formulations are quite useful because material parameters of most multiaxial criteria are derived from fatigue characteristics retrieved for pure axial loading and for pure torsion. Furthermore, this load combination belongs to the most often tested experimental setup in the multiaxial fatigue experiments. The formulas presented in [1] were then used e.g., by Papuga et al. [2] when deriving the material parameters of his two new multiaxial fatigue strength criteria.

One of the key parts of the analysis presented in [1] is the decision on how to cope with the description of the shear stress parameters on the evaluated plane. When some periodic load path is assessed, its projection onto the evaluated plane determines the shear stress path. If loading is proportional, the shear stress path is a simple line. Once loading gets non-proportional, the projection of the terminal point of the stress vector in time onto the evaluated plane becomes a two-dimensional shape. It is a closed object if loading is periodic, and it gets chaotic if random loading is imposed. Various multiaxial criteria then can use different strategies to detect the shear stress amplitude, and the mean shear stress (if this parameter is assessed at all). Papadopoulos et al. describe several options for treating this problem. The solutions using the scheme of the longest projection of the shear stress path or of its longest chord are rejected as obsolete or as ambiguous. The solution they mark as the optimum one is the minimum circumscribed circle (MCC) method based on circumscribing the smallest possible circle to the whole shear stress path (see also Figure 2 for illustration). To find this envelope, the authors propose to check all possible duos and triads of shear stress path points. Such an algorithm can result in a long computation time if the load path consists of more points to process. Weber et al. [5], Bernasconi [6] or recently Scalet [7] propose several optimization strategies to reach the solution of this problem quicker.

The calculation speed for the MCC problem is not the decisive issue, which is why some other authors proposed to adopt another strategy. Li et al. in [8] object that the MCC method cannot differentiate between the proportional and non-proportional load paths. Though the projection of the proportional load path onto the evaluated plane is a line and the projection of non-proportional loading is a two-dimensional shape, the same circle can encompass both. There is quite a firmly rooted expectation among some authors that non-proportional loading causes increased damage if compared to proportional loading, see e.g., [9] if the stress magnitudes are equal for both compared cases. The fact that the shear stress path for both variants can lead to the circumscribed circle of identical dimensions is the reason for proposals to change the strategy. Li et al. [8] propose to use the minimum circumscribed ellipse (MCE) instead, for which the shear stress amplitude is defined as the vector product of both semi-axes. Multiple procedures to define the right ellipse are defined, the summary of which is provided by Meggiolaro and Castro [9]. The iterative process to define the ellipse can however get even more complicated than the circle was, and this is the reason for further simplifications.

The principle of the maximum prismatic hull (MPH) was therefore defined [10]. As with the MCE principle, the shear stress amplitude is defined as the vector product of both semi-axes. Also this approach calls for the iterative process, while looking for the final MPH. Meggiolaro and Castro [11] thus proposed another simple measure—the Moment of Inertia (MOI) method, where the moments of inertia of the shear stress path is calculated as if the path was formed by a wire of a unit mass. Scalet in [12] comes with another approach—the principle of the convex hull enclosed on the stress path in the Ilyushin deviatoric 5D space.

All these concepts are based on the assumption that the minimum circumscribed circle method is wrong in the way the shear stress path signal is treated, because it does not differentiate sufficiently well between proportional loading and non-proportional loading. For non-proportional loading, the discussed modified definitions of the shear stress amplitude tend to result in higher values than the MCC variant would result. Such a claim should be supported by appropriate experimental data, but most of the validations done until now are inconclusive. Papuga et al. [13] explain that the way of treating the shear stress path on a specific plane need not have the expected impact on the critical plane criteria. If the detected maximum damage decides the critical plane there, the planes found critical for either MCE or for MCC concepts need not be the same. Second, the authors also highlight the question of the equivalent stress and of the norm used to decide, which type of loading—in-phase (IP) vs. out-of-phase (OOP)—is more damaging. Third, based on a sensitivity study they explain that the critical plane criteria of the maximum damage type lead to diminished damage for out-of-phase loading compared with in-phase loading. On the other hand, integral criteria and critical plane criteria of the maximum shear stress range type respond similarly for most materials except for brittle materials, where OOP loading causes higher damage compared with IP loading.

Within those sensitivity analyses, all multiaxial fatigue strength computations were performed while using the MCC concept. The number of other concepts invented to replace this method is large, so the analysis in this paper is extended to cover also those methods. There are not many such comparisons based on real experimental data and mostly they have been already cited here. They are often analyzed on relatively small data sets, into which also proportional load cases are included. The computational outputs for the proportional load cases do not differ for any of the stress path description methods, so these attempts to highlight the differences are weakened by the decision to include such cases. This is e.g., the case of Scalet [12] or Mamiya et al. [14]. These authors base their reasoning also on cases with different frequencies on different stress components. The difference in frequencies of concurrent loads however means that the load path gets more complicated. For that reason, some method of a load path decomposition to divide it into cycles should be involved in the computation routine. The fact that the authors skip this step and apply the stress path analysis method on its whole trajectory means that any evaluation of the output prediction quality need not focus on the right effect, and other uncovered ones can get unnoticed. Sahadi et al. [15], who discuss the prediction quality solely on their experiments, do not evaluate any non-proportional load path in their comparison of MCC and MPH concepts. This approach shows the limitation of using only own experimental data to uncover broader trends. The acceptance of also other sources of experiments is necessary. A survey over available experimental items in multiaxial high-cycle fatigue was recently delivered by Papuga et al. [16].

This paper provides an analytical formulation of the load path trajectory either when projected onto a specific plane or if transformed into the Ilyushin deviatoric space. The formulations extend the study by Papadopoulos et al. [1], who derived the analytical solution for general loading by axial load channel and by torsion load channel imposed independently by the testing machine. Here, however, the general load case is derived for the most complicated common multiaxial experiment—a pressurized hollow specimen loaded in addition axially and in torsion, with whichever phase shifts between the various harmonic load channels but with the same load periods. It should be noted that Papadopoulos et al. [1] referred explicitly to the bending and torsion load combination, while this paper refers to general axial loading causing axial normal stress. As regards the derived formulas, there is no contradiction, because the effect of non-constant stress distribution over the cross-section of a testing specimen is not covered at all.

These derivations for the first time prove that such projections are invariably ellipses, which can be analytically described. Thanks to this geometric feature, only three concepts of stress path processing (MCC, MCE and MOI) provide different estimates, while the other mentioned concepts (MPH or convex hull) result in the output identical with MCE. The paper further focuses on validating the output of these concepts, when integrated into six different multiaxial criteria of various types. Data items from the FatLim database of experiments [17] are primarily used for this validation. For the first time, the methods for processing the non-proportional stress paths combined with the multiaxial fatigue strength criteria are validated on a large set of experimental data supported with analytical derivation. Based on the results for these analyses, the optimum stress history processing methods are paired with the multiaxial criteria to reach the best estimates of fatigue strength.

## 2. Analytical Solution

**Figure 1:**
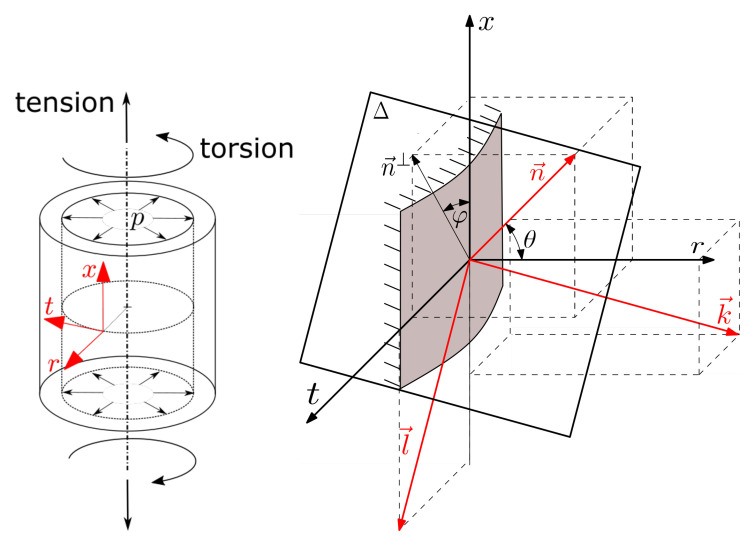
Used coordinate systems to describe the specimen orientation (**left**) and the evaluated Δ plane (**right**).

The paper focuses on the most often practically used multiaxial load configurations—a combination of acting axial, torsion and pressurizing load channels, which induce the stress tensor components:

(1) $$\Sigma \left(t\right)=\left[\begin{array}{ccc} \sigma_{x}\left(t\right) & \tau_{xt}\left(t\right) & 0\\ \tau_{xt}\left(t\right) & \sigma_{t}\left(t\right) & 0\\ 0 & 0 & \sigma_{r}\left(t\right) \end{array}\right]$$

i.e., the normal stress in *x* axial, *t* tangential and *r* radial directions and the shear stress in the xt direction (see also Figure 1). The other two shear stresses would be induced only if there is some further contact with the surface of the tested specimen. Such a scenario is unlikely for basic fatigue tests of unnotched smooth specimens. Most commonly, the load signal imposed in the high-cycle fatigue (HCF) experiments is harmonic, consisting of the *a* amplitude and *m* mean parts. The use of the harmonic signal is effective thanks to a simpler control of the machine drive, which would get complicated if any sharp peaks are induced in the signal. The complete stress tensor components look then:

(2) $$\Sigma \left(t\right)=\left[\begin{array}{ccc} \sigma_{x,a}\sin\left(\frac{2\pi t}{P}\right)+\sigma_{x,m} & \tau_{xt,a}\sin\left(\frac{2\pi t}{P}-\delta_{\tau }\right)+\tau_{xt,m} & 0\\ \tau_{xt,a}\sin\left(\frac{2\pi t}{P}-\delta_{\tau }\right)+\tau_{xt,m} & \sigma_{t,a}\sin\left(\frac{2\pi t}{P}-\delta_{t}\right)+\sigma_{t,m} & 0\\ 0 & 0 & \sigma_{r,a}\sin\left(\frac{2\pi t}{P}-\delta_{r}\right)+\sigma_{r,m} \end{array}\right]$$

Various phase shifts $\delta_{\tau }$, $\delta_{t}$ or $\delta_{r}$ are involved to make the solution more general, though the $\delta_{t}$ and $\delta_{r}$ phase shifts of tangential and radial stress signals, respectively, are usually identical, as both stress components are induced by the same pressurizing process. In all mathematical operations hereafter, it is assumed that all load channels act with the same load period *P*.

### 2.1. Stress Parameters on the Critical Plane

The two Euler angles θ and φ shown in Figure 1 define the orientation of the evaluated plane. Coordinates of its normal line are:

(3) $$\begin{array}{c} n_{x}=\sin\theta \cos\varphi \;n_{t}=\sin\theta \sin\varphi \;n_{r}=\cos\theta \end{array}$$

The normal stress on Δ plane is obtained from

(4) $$\begin{array}{c} N=\vec{n}\cdot \Sigma \cdot \vec{n} \end{array}$$

The mean value of the normal stress is derived from all time-independent addends of *N*:

(5) $$N_{m}=\sin^{2}\theta \left(\sigma_{x,m}\cos^{2}\varphi +\tau_{xt,m}\sin2\varphi +\sigma_{t,m}\sin^{2}\varphi \right)+\sigma_{r,m}\cos^{2}\theta$$

After several mathematical steps, the normal stress amplitude can be written as:

(6) $$\begin{array}{cc} N_{a}= & [{\left(\sigma_{x,a}\sin^{2}\theta \cos^{2}\varphi +\tau_{xt,a}\sin^{2}\theta \cos\delta_{\tau }\sin2\varphi +\sigma_{t,a}\sin^{2}\theta \cos\delta_{t}\sin^{2}\varphi +\sigma_{r,a}\cos^{2}\theta \cos\delta_{r}\right)}^{2}+\\ \; & +{\left(-\tau_{xt,a}\sin^{2}\theta \sin\delta_{\tau }\sin2\varphi -\sigma_{t,a}\sin^{2}\theta \sin\delta_{t}\sin^{2}\varphi -\sigma_{r,a}\sin\delta_{r}\cos^{2}\theta \right)}^{2}{]}^{\frac{1}{2}} \end{array}$$

To describe the course of the shear stress Δ on the evaluated plane, the coordinates nlk are used. Axes *l* and *k* are lying on Δ plane, to which *n* is the normal line. The unit vectors *l* a *k* can be written as:

(7) $$\begin{array}{c} l_{x}=-\sin\varphi \;l_{t}=\cos\varphi \;l_{r}=0 \end{array}$$

(8) $$\begin{array}{c} k_{x}=-\cos\theta \cos\varphi \;k_{t}=-\cos\theta \sin\varphi \;k_{r}=\sin\theta \end{array}$$

The shear stress on Δ plane can then be calculated from:

(9) $$\begin{array}{cc} C_{l} & =\vec{l}\cdot \vec{C}=\vec{l}\cdot \Sigma \cdot \vec{n} \end{array}$$

(10) $$\begin{array}{cc} C_{k} & =\vec{k}\cdot \vec{C}=\vec{k}\cdot \Sigma \cdot \vec{n} \end{array}$$

If the matrix multiplication is performed, we get to:

(11) $$C_{l}=-\frac{1}{2}\sigma_{x}\sin2\varphi \sin\theta +\tau_{xt}\sin\theta \cos2\varphi +\frac{1}{2}\sigma_{t}\sin\theta \sin2\varphi$$

(12) $$\begin{array}{cc} C_{k}= & -\frac{1}{2}\sigma_{x}\sin2\theta \cos^{2}\varphi -\frac{1}{2}\tau_{xt}\sin2\theta \sin2\varphi -\\ & -\frac{1}{2}\sigma_{t}\sin2\theta \sin^{2}\varphi +\frac{1}{2}\sigma_{r}\sin2\theta \end{array}$$

After some further mathematical manipulation, this record is obtained:

(13) $$\begin{array}{cc} C_{l}\left(t\right)= & f\sin\frac{2\pi t}{P}+g\cos\frac{2\pi t}{P}+\\ & +\left(-\frac{1}{2}\sigma_{x,m}\sin2\varphi +\tau_{xt,m}\cos2\varphi +\frac{1}{2}\sigma_{t,m}\sin2\varphi \right)\sin\theta \end{array}$$

(14) $$\begin{array}{cc} C_{k}\left(t\right)= & p\sin\frac{2\pi t}{P}+q\cos\frac{2\pi t}{P}-\\ & -\frac{1}{2}\left(\sigma_{x,m}\cos^{2}\varphi +\tau_{xt,m}\sin2\varphi +\sigma_{t,m}\sin^{2}\varphi -\sigma_{r,m}\right)\sin2\theta \end{array}$$

where functions *f*, *g*, *p* and *q* are:

(15) $$f=\left(-\frac{1}{2}\sigma_{x,a}\sin2\varphi +\tau_{xt,a}\cos\delta_{\tau }\cos2\varphi +\frac{1}{2}\sigma_{t,a}\cos\delta_{t}\sin2\varphi \right)\sin\theta$$

(16) $$g=\left(-\tau_{xt,a}\sin\delta_{\tau }\cos2\varphi -\frac{1}{2}\sigma_{t,a}\sin\delta_{t}\sin2\varphi \right)\sin\theta$$

(17) $$\begin{array}{cc} p=-\frac{1}{2}( & \sigma_{x,a}\cos^{2}\varphi +\tau_{xt,a}\cos\delta_{\tau }\sin2\varphi +\sigma_{t,a}\cos\delta_{t}\sin^{2}\varphi -\\ & -\sigma_{r,a}\cos\delta_{r})\sin2\theta \end{array}$$

(18) $$q=\frac{1}{2}\left(\tau_{xt,a}\sin\delta_{\tau }\sin2\varphi +\sigma_{t,a}\sin\delta_{t}\sin^{2}\varphi -\sigma_{r,a}\sin\delta_{r}\right)\sin2\theta$$

Equations (13) and (14) correspond to parametric formulas describing an ellipse, which is the load path projection of the general load history (Equation (2)) onto Δ plane. Its centre is derived from time-independent parts of the formulas:

(19) $$\begin{array}{c} C_{l,m}=\left(-\frac{1}{2}\sigma_{x,m}\sin2\varphi +\tau_{xt,m}\cos2\varphi +\frac{1}{2}\sigma_{t,m}\sin2\varphi \right)\sin\theta \end{array}$$

(20) $$\begin{array}{c} C_{k,m}=-\frac{1}{2}\left(\sigma_{x,m}\cos^{2}\varphi +\tau_{xt,m}\sin2\varphi +\sigma_{t,m}\sin^{2}\varphi -\sigma_{r,m}\right)\sin2\theta \end{array}$$

The centre of either the MCE or the MCC is therefore computed from these values as:

(21) $$C_{m}=\sqrt{C_{l,m}^{2}+C_{k,m}^{2}}$$

Semi-axes of the ellipse stress path are:

(22) $$a,b=\sqrt{\frac{f^{2}+g^{2}+p^{2}+q^{2}}{2}\pm \sqrt{{\left(\frac{f^{2}+g^{2}+p^{2}+q^{2}}{2}\right)}^{2}-{\left(fq-gp\right)}^{2}}}$$

The smallest circumscribed circle has its radius equal to the longer semi-axis of the ellipse:

(23) $$\begin{array}{c} C_{a,MCC}=\sqrt{\frac{f^{2}+g^{2}+p^{2}+q^{2}}{2}+\sqrt{{\left(\frac{f^{2}+g^{2}+p^{2}+q^{2}}{2}\right)}^{2}-{\left(fq-gp\right)}^{2}}} \end{array}$$

The smallest circumscribed ellipse is identical with the stress path shape, and thus the shear stress amplitude can be derived from semi-axes written above in Equation (22) as their vector product:

(24) $$C_{a,MCE}=\sqrt{a^{2}+b^{2}}=\sqrt{f^{2}+g^{2}+p^{2}+q^{2}}$$

**Figure 2:**
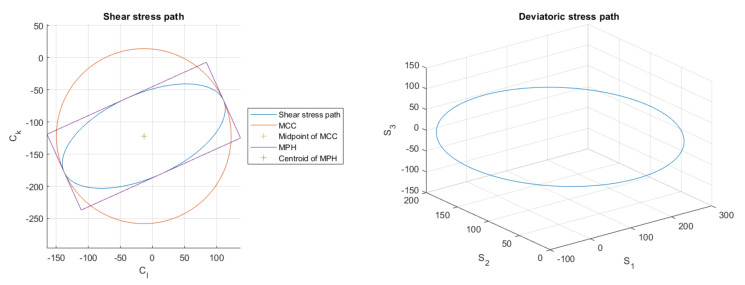
Shear stress path on the critical plane and the enveloping entities (**left**). Depiction of the same stress path in the 3D-deviatoric space to which the Ilyushin deviatoric space degenerates for the general load case described by Equation (2) (see **right**). These load paths correspond to the experiment by Troost et al. [18] with $\sigma_{x,a}=223.6$ MPa, $\sigma_{x,m}=255.0$ MPa, $\sigma_{t,a}=167.0$ MPa, $\sigma_{t,m}=210.8$ MPa, $\tau_{xt,a}=111.8$ MPa, $\delta_{\tau }=90$ deg, $\delta_{t}=180$ deg.

To illustrate the output stress path, a quite complex load case TAK10 was chosen, see Figure 2, where the stress path projection onto the evaluated plane is depicted left. The most important outcome of this section is that whichever the phase shifts are on the four individual stress channels if all acting loads are harmonic and of identical frequency, the final shear stress path is the ellipse. Parameters of this ellipse can be readily computed via Equations (21) and (24). Due to the simple geometric shape of the stress path, the output of the maximum prismatic hull or of the convex hull will be identical to the MCE output. Use of these analytical formulas can save substantial computational time if the stated conditions on load types are met.

The shear stress path description by Equations (13) and (14) is also used to describe the parametric formula $P_{C}\left(t\right)=\left(C_{l}\left(t\right),C_{k}\left(t\right)\right)$, along which the curve integral is computed for the MOI method. Its derivation ${\dot{P}}_{C}\left(t\right)=\left({\dot{C}}_{l}\left(t\right),{\dot{C}}_{k}\left(t\right)\right)$ is necessary:

(25) $$\begin{array}{c} {\dot{C}}_{l}\left(t\right)=f\frac{2\pi }{P}\cos\frac{2\pi t}{P}-g\frac{2\pi }{P}\sin\frac{2\pi t}{P} \end{array}$$

(26) $$\begin{array}{c} {\dot{C}}_{k}\left(t\right)=p\frac{2\pi }{P}\cos\frac{2\pi t}{P}-q\frac{2\pi }{P}\sin\frac{2\pi t}{P} \end{array}$$

The perimeter of the elliptic shear stress path is defined:

(27) $$p_{C}=\oint dp_{C}=\int_{0}^{P}\left|\right|{\dot{P}}_{C}\left(t\right)\left|\right|dt=\int_{0}^{P}\sqrt{{\dot{C}}_{l}{\left(t\right)}^{2}+{\dot{C}}_{k}{\left(t\right)}^{2}}dt$$

and the mean value of the shear stress is given by:

(28) $$\begin{array}{c} C_{l,m}=\frac{1}{p_{C}}\oint C_{l}dp_{C}=\frac{1}{p_{C}}\int_{0}^{P}C_{l}\sqrt{{\dot{C}}_{l}{\left(t\right)}^{2}+{\dot{C}}_{r}{\left(t\right)}^{2}}dt \end{array}$$

(29) $$\begin{array}{c} C_{k,m}=\frac{1}{p_{C}}\oint C_{k}dp_{C}=\frac{1}{p_{C}}\int_{0}^{P}C_{k}\sqrt{{\dot{C}}_{l}{\left(t\right)}^{2}+{\dot{C}}_{k}{\left(t\right)}^{2}}dt \end{array}$$

(30) $$\begin{array}{c} C_{m}=\sqrt{C_{l,m}^{2}+C_{k,m}^{2}} \end{array}$$

The polar moment of inertia:

(31) $$\begin{array}{cc} I_{p} & =\frac{1}{p_{C}}\oint \left({\left(C_{l}\left(t\right)-C_{l,m}\right)}^{2}+{\left(C_{k}\left(t\right)-C_{k,m}\right)}^{2}\right)dp_{C}\\ & =\frac{1}{p_{C}}\int_{0}^{P}\left({\left(C_{l}\left(t\right)-C_{l,m}\right)}^{2}+{\left(C_{k}\left(t\right)-C_{k,m}\right)}^{2}\right)\sqrt{{\dot{C}}_{l}{\left(t\right)}^{2}+{\dot{C}}_{k}{\left(t\right)}^{2}}dt \end{array}$$

is used to define the shear stress amplitude based on the condition that the shear stress amplitude should be equal to one half of the abscissa corresponding to the stress path in IP loading:

(32) $$C_{a,MOI}=\sqrt{3I_{p}}$$

### 2.2. Stress Parameters in the Ilyushin Deviatoric Space

Typically, the multiaxial criteria based on the load path analysis in the Ilyushin deviatoric space are based on combining the hydrostatic stress with the second invariant of the stress deviator. Hydrostatic stress equals:

(33) $$\begin{array}{cc} \sigma_{H}= & \frac{1}{3}\left(\sigma_{x}+\sigma_{t}+\sigma_{r}\right) \end{array}$$

Its mean value is set from the time-independent terms:

(34) $$\sigma_{H,m}=\frac{1}{3}\left(\sigma_{x,m}+\sigma_{t,m}+\sigma_{r,m}\right)$$

The amplitude of hydrostatic stress is obtained when the mean value is subtracted from the total:

(35) $$\sigma_{H,a}=\frac{1}{3}\sqrt{{\left(\sigma_{x,a}+\sigma_{t,a}\cos\delta_{t}+\sigma_{r,a}\cos\delta_{r}\right)}^{2}+{\left(-\sigma_{t,a}\sin\delta_{t}-\sigma_{r,a}\sin\delta_{r}\right)}^{2}}$$

The stress deviator is derived from the stress tensor and from hydrostatic stress:

(36) $$s=\Sigma -\sigma_{H}I=\left[\begin{array}{ccc} \frac{2\sigma_{x}-\sigma_{t}-\sigma_{r}}{3} & \tau_{xt} & 0\\ \tau_{xt} & \frac{2\sigma_{t}-\sigma_{x}-\sigma_{r}}{3} & 0\\ 0 & 0 & \frac{2\sigma_{r}-\sigma_{x}-\sigma_{t}}{3} \end{array}\right]$$

where I is the second-order unit tensor. To obtain the second invariant of the stress deviator $\sqrt{J_{2}}$ needed for the criteria using this type of solution, it is useful to transform the stress deviator s into the vector $\vec{S}$ of these components in a 5-dimensional space:

(37) $$\begin{array}{c} S_{1}=\frac{\sqrt{3}}{2}s_{x}\;S_{2}=\frac{1}{2}\left(s_{t}-s_{r}\right)\;S_{3}=s_{xt} \end{array}$$

(38) $$\begin{array}{c} S_{4}=s_{xr}\;S_{5}=s_{tr} \end{array}$$

The second key stress parameter in multiaxial criteria processed in the Ilyushin deviatoric space—the square root of the second invariant of the stress deviator—is obtained from:

(39) $$\sqrt{J_{2}}=\sqrt{\frac{1}{2}s\cdot s}=\sqrt{\vec{S}\cdot \vec{S}}$$

If the transformed coordinates are used:

(40) $$\begin{array}{c} S_{1}=\frac{\sqrt{3}}{2}\cdot \frac{2\sigma_{x}-\sigma_{t}-\sigma_{r}}{3} \end{array}$$

(41) $$\begin{array}{c} S_{2}=\frac{1}{2}\left(\sigma_{t}-\sigma_{r}\right) \end{array}$$

(42) $$\begin{array}{c} S_{3}=\tau_{xt} \end{array}$$

(43) $$\begin{array}{c} S_{4}=0 \end{array}$$

(44) $$\begin{array}{c} S_{5}=0 \end{array}$$

This effectively means that only 3-parametric space is sufficient to describe the load path given by Equation (2) in its entirety. If the functions of individual stress tensor components are used, the final formulas of individual non-zero components can be obtained:

(45) $$\begin{array}{c} S_{1}=S_{1s}\sin\frac{2\pi t}{P}+S_{1c}\cos\frac{2\pi t}{P}+S_{1,m} \end{array}$$

(46) $$\begin{array}{c} S_{2}=S_{2s}\sin\frac{2\pi t}{P}+S_{2c}\cos\frac{2\pi t}{P}+S_{2,m} \end{array}$$

(47) $$\begin{array}{c} S_{3}=S_{3s}\sin\frac{2\pi t}{P}+S_{3c}\cos\frac{2\pi t}{P}+S_{3,m} \end{array}$$

in which the functions $S_{1s}$, $S_{1c}$, $S_{2s}$, $S_{2c}$, $S_{3s}$ a $S_{3c}$ are:

(48) $$\begin{array}{c} S_{1s}=\frac{1}{\sqrt{3}}\left(\sigma_{x,a}-\frac{\sigma_{t,a}}{2}\cos\delta_{t}-\frac{\sigma_{r,a}}{2}\cos\delta_{r}\right) \end{array}$$

(49) $$\begin{array}{c} S_{1c}=\frac{1}{2\sqrt{3}}\left(\sigma_{t,a}\sin\delta_{t}+\sigma_{r,a}\sin\delta_{r}\right) \end{array}$$

(50) $$\begin{array}{c} S_{2s}=\frac{1}{2}\left(\sigma_{t,a}\cos\delta_{t}-\sigma_{r,a}\cos\delta_{r}\right) \end{array}$$

(51) $$\begin{array}{c} S_{2c}=\frac{1}{2}\left(-\sigma_{t,a}\sin\delta_{t}+\sigma_{r,a}\sin\delta_{r}\right) \end{array}$$

(52) $$\begin{array}{c} S_{3s}=\tau_{xt,a}\cos\delta_{\tau } \end{array}$$

(53) $$\begin{array}{c} S_{3c}=-\tau_{xt,a}\sin\delta_{\tau } \end{array}$$

The formulas in Equations (45)–(47) describe the parametric definition of an ellipse in the $E_{3}$ space. Its center is derived from the time-independent parts:

(54) $$S_{1,m}=\frac{1}{\sqrt{3}}\left(\sigma_{x,m}-\frac{\sigma_{t,m}}{2}-\frac{\sigma_{r,m}}{2}\right)\;S_{2,m}=\frac{1}{2}\left(\sigma_{t,m}-\sigma_{r,m}\right)\;S_{3,m}=\tau_{xt,m}$$

and so the mean value $\sqrt{J_{2,m}}$ is computed from:

(55) $$\sqrt{J_{2,m}}=\sqrt{S_{1,m}^{2}+S_{2,m}^{2}+S_{3,m}^{2}}$$

The length of both ellipse semi-axes is defined:

(56) $$\begin{array}{cc} a,b= & [\frac{S_{1s}^{2}+S_{1c}^{2}+S_{2s}^{2}+S_{2c}^{2}+S_{3s}^{2}+S_{3c}^{2}}{2}\pm \\ & [{\left(\frac{S_{1s}^{2}+S_{1c}^{2}+S_{2s}^{2}+S_{2c}^{2}+S_{3s}^{2}+S_{3c}^{2}}{2}\right)}^{2}-({\left(S_{1s}S_{2c}-S_{1c}S_{2s}\right)}^{2}\\ & +{\left(S_{1s}S_{3c}-S_{1c}S_{3s}\right)}^{2}+{\left(S_{2s}S_{3c}-S_{2c}S_{3s}\right)}^{2}){]}^{\frac{1}{2}}{]}^{\frac{1}{2}} \end{array}$$

The minimum circumscribed ball enveloping the elliptic load path in the $E_{3}$ space has the identical centre as the ellipse and its radius is equal to the longer semi-axis of the ellipse. The minimum circumscribed ellipsoid expected in the Ilyushin deviatoric space thus degenerates for the load case combination given by Equation (2) into the ellipse located in the $E_{3}$ space. The amplitude $\sqrt{J_{2,a}}$ in the MCE concept is therefore computed from the vector product of all ellipse semi-axes:

(57) $$\sqrt{J_{2,a}}=\sqrt{a^{2}+b^{2}}=\sqrt{S_{1s}^{2}+S_{1c}^{2}+S_{2s}^{2}+S_{2c}^{2}+S_{3s}^{2}+S_{3c}^{2}}$$

The fact that, for the given composition of load channels and harmonic loading (see Equation (2), the load path when transformed into the Ilyushin deviatoric space results in an ellipse drawn in 3D space is a great simplification of the seemingly complicated reality. This load path can be well-characterised via the parametric formulation, and the ellipse parameters can be computed analytically. This largely simplifies and shortens the necessary computations of various multiaxial fatigue strength criteria.

In the MOI method, Equations (45)–(47) define the parametric description of the curve $P_{S}\left(t\right)$, along which the integration occurs. To calculate the curve integral, its derivation ${\dot{P}}_{S}\left(t\right)=\left({\dot{S}}_{1}\left(t\right),{\dot{S}}_{2}\left(t\right),{\dot{S}}_{3}\left(t\right)\right)$ is necessary:

(58) $$\begin{array}{c} {\dot{S}}_{1}\left(t\right)=S_{1s}\frac{2\pi }{P}\cos\frac{2\pi t}{P}-S_{1c}\frac{2\pi }{P}\sin\frac{2\pi t}{P} \end{array}$$

(59) $$\begin{array}{c} {\dot{S}}_{2}\left(t\right)=S_{2s}\frac{2\pi }{P}\cos\frac{2\pi t}{P}-S_{2c}\frac{2\pi }{P}\sin\frac{2\pi t}{P} \end{array}$$

(60) $$\begin{array}{c} {\dot{S}}_{3}\left(t\right)=S_{3s}\frac{2\pi }{P}\cos\frac{2\pi t}{P}-S_{3c}\frac{2\pi }{P}\sin\frac{2\pi t}{P} \end{array}$$

(61) $$p_{S}=\oint dp_{S}=\int_{0}^{P}\left|\right|{\dot{P}}_{S}\left(t\right)\left|\right|dt=\int_{0}^{P}\sqrt{{\dot{S}}_{1}{\left(t\right)}^{2}+{\dot{S}}_{2}{\left(t\right)}^{2}+{\dot{S}}_{3}{\left(t\right)}^{2}}dt$$

The mean value $\sqrt{J_{2,m}}$ is computed from:

(62) $$\begin{array}{c} S_{1,m}=\frac{1}{p_{S}}\oint S_{1}dp_{S}=\frac{1}{p_{S}}\int_{0}^{P}S_{1}\sqrt{{\dot{S}}_{1}{\left(t\right)}^{2}+{\dot{S}}_{2}{\left(t\right)}^{2}+{\dot{S}}_{3}{\left(t\right)}^{2}}dt \end{array}$$

(63) $$\begin{array}{c} S_{2,m}=\frac{1}{p_{S}}\oint S_{2}dp_{S}=\frac{1}{p_{S}}\int_{0}^{P}S_{2}\sqrt{{\dot{S}}_{1}{\left(t\right)}^{2}+{\dot{S}}_{2}{\left(t\right)}^{2}+{\dot{S}}_{3}{\left(t\right)}^{2}}dt \end{array}$$

(64) $$\begin{array}{c} S_{3,m}=\frac{1}{p_{S}}\oint S_{3}dp_{S}=\frac{1}{p_{S}}\int_{0}^{P}S_{3}\sqrt{{\dot{S}}_{1}{\left(t\right)}^{2}+{\dot{S}}_{2}{\left(t\right)}^{2}+{\dot{S}}_{3}{\left(t\right)}^{2}}dt \end{array}$$

(65) $$\begin{array}{c} \sqrt{J_{2,m}}=\sqrt{S_{1,m}^{2}+S_{2,m}^{2}+S_{3,m}^{2}} \end{array}$$

The amplitude of the stress tensor deviator is again defined:

(66) $$\sqrt{J_{2,a}}=\sqrt{3I_{p}}$$

while the polar moment of inertia is:

(67) $$\begin{array}{cc} I_{p} & =\frac{1}{p_{S}}\oint \left({\left(S_{1}\left(t\right)-S_{1,m}\right)}^{2}+{\left(S_{2}\left(t\right)-S_{2,m}\right)}^{2}+{\left(S_{3}\left(t\right)-S_{3,m}\right)}^{2}\right)dp_{S}\\ & =\frac{1}{p_{S}}\int_{0}^{P}\left({\left(S_{1}\left(t\right)-S_{1,m}\right)}^{2}+{\left(S_{2}\left(t\right)-S_{2,m}\right)}^{2}+{\left(S_{3}\left(t\right)-S_{3,m}\right)}^{2}\right)\\ & \sqrt{{\dot{S}}_{1}{\left(t\right)}^{2}+{\dot{S}}_{2}{\left(t\right)}^{2}+{\dot{S}}_{3}{\left(t\right)}^{2}}dt \end{array}$$

## 3. Sensitivity Study

The stress path described in Equation (2) forms the ellipse in both analyzed 2D and 3D spaces. It can be mathematically described, so there is no need for any iterative analysis in such a case. As a consequence of the elliptic shape, the multitude of approaches to the load path analysis described in the Introduction section considerably lessens as regards the variability of the output. The convex hull approach by Scalet [12], and the maximum prismatic hull approach [10] will provide the output identical to the minimum circumscribed ellipse. For out-of-phase loading, all these approaches generate the amplitude parameter larger than the one obtained for the minimum circumscribed circle approach. The MCC method results in its value equal to the longer semi-axis of the ellipse, while the MCE (and all other mentioned methods based on enveloping entities) will provide this value increased thanks to involving also the shorter semi-axis in the final vector product. The authors did not proceed to derive the complete analytical formula also for the MOI method by Meggiolaro and Castro [11]. The solution seems to be very complicated, and its output as presented hereafter is far less promising and it does not seem it deserves to invest more effort.

A more practical comparison of the output of individual stress path description methods is thus desirable. Papuga et al. designed in [13] a special sensitivity study, which allowed them to assess the response of various multiaxial fatigue strength criteria to the condition of a varying phase shift effect. Three different fictive materials were established to simulate a potentially different material response. They differ by the most important multiaxial characteristics, the fatigue strength ratio κ, which can be computed:

(68) $$\kappa =\frac{s_{-1}}{t_{-1}}$$

from the fatigue strength in fully reversed axial loading $s_{-1}$ and from the fatigue strength in fully reversed torsion $t_{-1}$. Brittle material with κ = 1.07, ductile material with κ = 1.58 and extra-ductile material with κ = 1.82 were proposed. For these three material setups, load cases described by four different load ratios $r_{\sigma }$:

(69) $$r_{\sigma }=\frac{\sigma_{x,a}}{\tau_{xt,a}}$$

between axial stress and shear stress were proposed. The response of the checked multiaxial fatigue strength prediction criteria to various load cases differing by the phase shift between both load channels was analyzed. The phase shift varied from 0 deg to 180 deg. The graphs showed the fatigue strength response is symmetrical around 90 deg.

The same procedure was also processed here. Whereas [13] focused solely on the MCC approach, here the MCE and MOI approaches were tested additionally. To check the typical response to various concepts of the multiaxial fatigue strength analysis, six different multiaxial criteria were chosen to compute the equivalent stress amplitude $\sigma_{eq,a}$. Two criteria look for the critical plane defined as the plane of the maximum damage (or equivalent stress)—these are the Papuga PCRN method (acronym for Papuga Critical plane method in Revised Newer version, see [19]):

(70) $$\sigma_{eq,a}=\max_{\varphi ,\theta }\left(\sqrt{a_{PC}C_{a}\left(C_{a}+c_{PC}C_{m}\right)+b_{PC}\sqrt{N_{a}(N_{a}+d_{PC}N_{m}}}\right)$$

and the Findley method (hereafter marked FIN, see [20] or [21]):

(71) $$\sigma_{eq,a}=\max_{\varphi ,\theta }\left(a_{F}C_{a}+b_{F}N_{max}\right)$$

In all Equations (70)–(75), the variously indexed parameters *a*, *b*, *c* or *d* represent material parameters derived from basic uniaxial load conditions. The parameters *a* and *b* are usually obtained from fatigue strengths at fully reversed axial loading and at fully reversed torsion loading. To derive parameters *c* and *d*, e.g., repeated axial loading and repeated torsion tests are required in addition to provide the necessary fatigue strengths.

Another critical plane concept—the critical plane selected as the plane of maximum shear stress range—was evaluated on the example of the Matake criterion (shortened to MAT, [21,22]):

(72) $$\sigma_{eq,a}=a_{M}C_{a}+b_{M}N_{max}$$

The request to find the maximum shear stress range first to locate the critical plane can be solved in different ways. It can be defined as the longest shear stress path projection onto a specific direction on the evaluated plane. However, it can also be the $C_{a}$ parameter obtained by the MCC or MCE schemes. If the final stress path is elliptical, the difference between the maximum projection and MCC will be zero. MCE will definitely reach other results while taking into account also the shorter semi-axis of the ellipse. The question was solved at last in such a way that the $C_{a,MCC}$ and $C_{a,MCE}$ shear stress amplitudes are used to define the critical planes for the Matake criterion.

The Papuga PIN criterion (acronym for Papuga Integral criterion in the Newer version) [23]:

(73) $$\sigma_{eq,a}=\sqrt{\frac{1}{4\pi }\int_{\varphi =0}^{2\pi }\int_{\theta =0}^{\pi }a_{PI}C_{a}\left(C_{a}+c_{PI}C_{m}\right)+b_{PI}N_{a}\left(N_{a}+d_{PI}N_{m}\right)\sin\theta d\theta d\varphi }$$

and the Liu and Zenner method (LZ method, [24] or [21]):

(74) $$\sigma_{eq,a}=\sqrt{\frac{15}{8\pi }\int_{\varphi =0}^{2\pi }\int_{\theta =0}^{\pi }a_{LZ}C_{a}^{2}\left(1+c_{LZ}C_{m}^{2}\right)+b_{LZ}N_{a}^{2}\left(1+d_{LZ}N_{m}\right)\sin\theta d\theta d\varphi }$$

represent the integral multiaxial criteria. The last type of multiaxial criteria is the solution using the Ilyushin deviatoric space. The Crossland method (CROSS, [25] or [21]) was chosen here:

(75) $$\sigma_{eq,a}=a_{C}\sqrt{J_{2,a}}+b_{C}\sigma_{H,max}$$

If the chosen stress combination processed in the multiaxial fatigue strength criterion gives the equivalent stress amplitude equal to the given fatigue strength in fully reversed axial loading $s_{-1}$, the specimen should break. Proximity to such a state is described by the fatigue index FI:

(76) $$FI=\frac{\sigma_{eq,a}}{s_{-1}}$$

**Figure 3:**
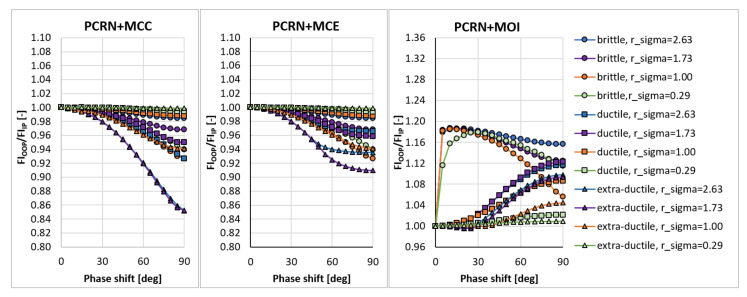
Comparison of the output of the PCRN critical plane method of the maximum damage type.

**Figure 4:**
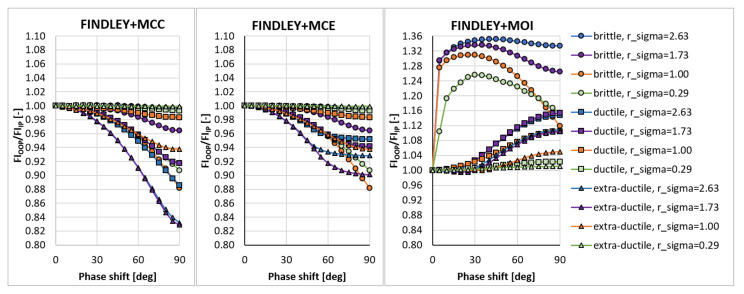
Comparison of the output of the Findley critical plane method of the maximum damage type.

**Figure 5:**
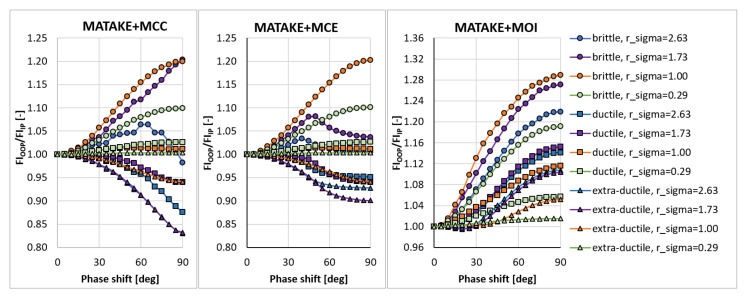
Comparison of the output of the Matake critical plane method of the maximum shear stress range type.

**Figure 6:**
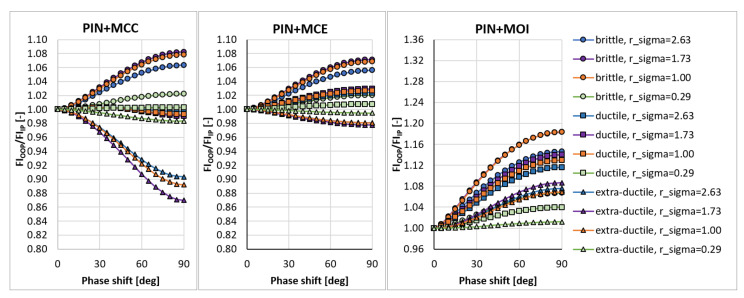
Comparison of the output of the PIN integral method.

**Figure 7:**
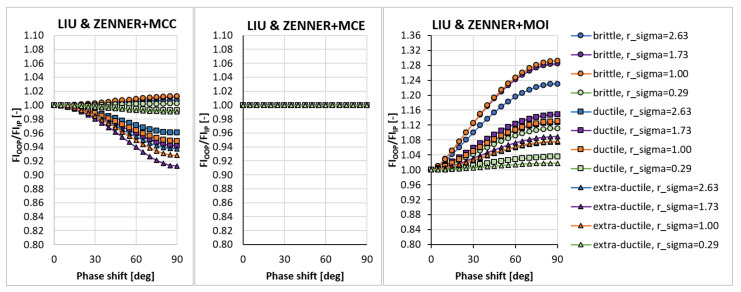
Comparison of the output of the Liu and Zenner integral method.

**Figure 8:**
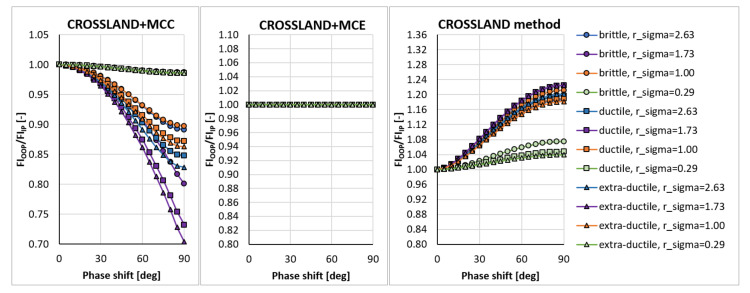
Comparison of the output of the Crossland method.

In the sensitivity study presented here, the acting stress levels at each load combination described by $r_{\sigma }$ were set in such a way that $FI_{IP}$ close to 1.0 was obtained for the PCRN criterion when the phase shift $\delta_{\tau }$ was zero (in-phase loading, IP). Other phase shifts with the angular step of 5 deg between individual variants (i.e., out-of-phase loading, OOP, $\delta_{\tau }\neq 0$ deg) were then evaluated to compute $FI_{OOP}$ with the magnitudes of acting stresses kept identical to the in-phase loading configuration. The output for these various phase shifts was normalized by $FI_{IP}$ to show the change in the response of each criterion. These trends can be found in Figure 3, Figure 4, Figure 5, Figure 6, Figure 7 and Figure 8. For in-phase loading ($\delta_{\tau }=0$ deg), the response of the $FI_{OOP}/FI_{IP}$ ratio must be at 1.00. When the curve goes down at higher phase shifts, it means that the estimated equivalent stress is lower for the specified phase shift, than it would be for IP loading, though the stress levels at both load channels remain the same for IP and OOP variants. This trend can also be interpreted as a damage decrease invoked by de-phasing the load channels (when the component stress maximums do not coincide in time). Some of the criteria (Figure 5 or Figure 6) also show the opposite trend—de-phasing leads to increasing the damage. This trend is apparent for brittle material there.

For most criteria, the result trends for the MCE and MCC concepts do not differ much for cases with sufficiently big shear stresses (curves with orange and green symbols in Figure 3, Figure 4, Figure 5, Figure 6, Figure 7 and Figure 8). A bigger variability of the response of MCC and MCE methods can be observed for the cases with prevalent axial stress. The Papuga PCRN criterion (Figure 3) and the Findley criterion (Figure 4) are of a similar type—they are both critical plane criteria, where the critical plane is found as the plane with the maximum damage (i.e., maximum equivalent stress). Their response in the MCC configuration is relatively similar, though the Findley method exhibits the more downward trend of curves for the prevalent axial stress. The change occurring when the MCE concept is used affects above all the curves of extra-ductile material, which are shifted to much bigger equivalent stresses in the out-of-phase load cases. Both these critical plane criteria show a doubtful response for the MOI approach since there is an extremely abrupt change of the equivalent stress response between $\delta_{\tau }=0$ deg and $\delta_{\tau }=5$ deg for brittle materials. The MOI behaves differently if compared with MCC and MCE approach—almost all load ratios and all materials respond to de-phasing by increasing the equivalent stress (or the damage caused).

The Matake criterion (Figure 5) also uses the critical plane concept, but the critical plane is selected as the plane, on which the maximum shear stress range is found. This condition substantially modifies the response of the criterion. The most important difference to the PCRN criterion and to the Findley criterion is that the increase of damage caused by de-phasing can be found for brittle material. The application of MCE modifies the response for the cases with prevalent axial stress above all. The MOI approach avoids the questionable behaviour of brittle materials documented by the PCRN and Findley methods, but otherwise, the trend of de-phasing increasing the damage for almost all cases can be observed here as well.

There are two representatives of the integral methods—the Papuga PIN method (Figure 6) and the Liu and Zenner method (Figure 7). While the Liu and Zenner solution shows a limited variability of the equivalent fatigue strength response to the phase shift, the PIN method spans over the 0.87-–1.08 period for different load cases and different materials. This evaluation concerns the output of the MCC variant—the variability of the output of the PIN criterion decreases, once MCE is applied, while its primary effect can be seen in the change for extra-ductile material. The MOI concept responds again in the same way as documented previously—any curve corresponds to increased damage once a non-zero phase shift is invoked between both load channels. This behaviour of MOI can be found quite universal for all methods hereafter, and only its magnitude differs.

A very extreme change when switching from the MCC concept to the MCE concept can be observed by Liu and Zenner integral method and by the Crossland method. If MCE is applied, the phase shift stops to play any role, and all curves depicted in Figure 8 for the MCC variant change to horizontal lines at $FI_{OOP}/FI_{IP}=1.0$ for the MCE. MOI again shows the same trend as previously. In all cases, the MOI concept results in causing bigger damage (or equivalent stress) for the OOP case, than would IP case induce. Because this behaviour quite strongly contradicts the behaviour of MCC and MCE concepts, it should be easily determined, which of those concepts is more realistic—if they are compared with real experimental results, either MCC will be too non-conservative, or MOI will be extremely conservative.

The explanation of the manifested insensitivity of the Crossland criterion to the phase shift between axial and shear stress signals (see Figure 8) detected for the MCE variant of the stress path analysis can be simply proven. The formulas in Equations (48)–(53) reduce for this type of loading to

(77) $$\begin{array}{c} S_{1s}=\frac{\sigma_{x,a}}{\sqrt{3}} \end{array}$$

(78) $$\begin{array}{c} S_{1c}=S_{2s}=S_{2c}=0 \end{array}$$

(79) $$\begin{array}{c} S_{3s}=\tau_{xt,a}\cos\delta_{\tau } \end{array}$$

(80) $$\begin{array}{c} S_{3c}=-\tau_{xt,a}\sin\delta_{\tau }. \end{array}$$

The amplitude of the square root of the second invariant of deviatoric stress thus results in:

(81) $$\sqrt{J_{2,a}}=\sqrt{S_{1s}^{2}+S_{3s}^{2}+S_{3c}^{2}}=\sqrt{\frac{\sigma_{x,a}^{2}}{3}+\tau_{xt,a}^{2}}$$

This formula is independent of $\delta_{\tau }$. Logically, the same independence on the phase shift will be observed for MCE also by other multiaxial fatigue strength criteria using $\sqrt{J_{2,a}}$—the Sines criterion [26] or the Kakuno-Kawada criterion [27].

## 4. Validation on Experimental Data

### 4.1. AMSD25 Set

The sensitivity study discussed above has shown some trends in the change of the response of the multiaxial fatigue strength criteria, when the original MCC method of load path description is changed to the MCE method or to the MOI method. The difference is observed above all for out-of-phase loading cases, for extra-ductile materials and for load cases with prevalent axial stresses over shear stress—for these cases the MCE concept predicts higher equivalent stress than the MCC variant. The only exceptions are the Matake method, where the selection of the critical plane is based on the value of the maximum shear stress range, i.e., a value independent from using MCC or MCE schemes, and the Liu and Zenner method, where brittle material results in equivalent stresses s lightly lower for the MCE method than for MCC. The MOI concept results in the wholly different response for all evaluated multiaxial fatigue strength criteria—de-phasing causes higher damage compared with the in-phase case of identical stress on both load channels for any of twelve tested configurations of materials and stress ratios.

All these documented changes in the fatigue prediction response describe behaviour of specific computational methods, which are based on a combination of the multiaxial fatigue strength criterion and of the stress path description. The output of the previous section does not relate to reality—the relation between the observed behaviour of the calculation method and the real behaviour of the material was not evaluated at all. To validate the proposed combinations, the experimental set AMSD25 proposed by Papuga et al. in [17] is used. This test set comprises 57 experimental load cases obtained on different materials by different teams [28,29,30,31,32,33,34,35,36,37,38,39,40,41,42,43]. These test items were selected from the original FatLim test set of 282 items (see also [16]) from the comparison of fatigue strength estimation results of 18 different multiaxial fatigue strength criteria. For the AMSD25 test set, those items for which the various tested multiaxial fatigue strength criteria provide too imprecise or too contradicting estimates were chosen [17].

**Figure 9:**
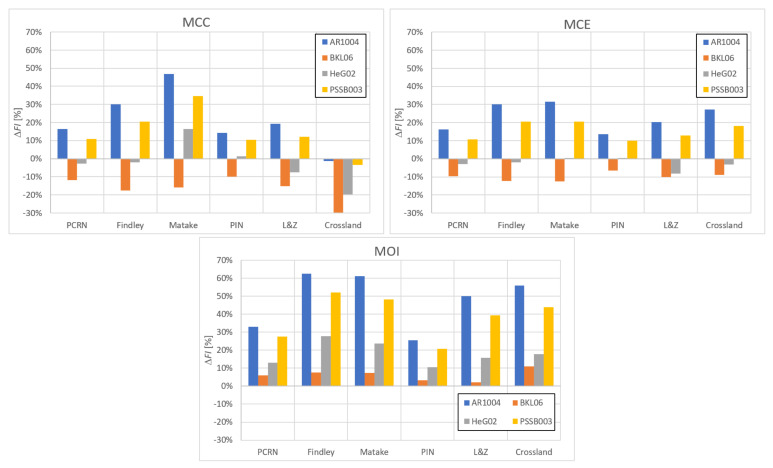
Results of applying the chosen multiaxial fatigue strength criteria to the out-of-phase experiments without any mean stresses from the AMSD25 test set.

In reality, the difference between various methods of assessing the stress path is observable only for out-of-phase cases. This condition comes true for only 11 items from the whole AMSD25 set of 57 items. From these test cases, only four are without any mean stresses involved, and thus the mean stress effect cannot affect the output of individual methods as regards the prediction quality. For these four test cases (inputs for which are shown in Table 1), the output of the fatigue index error ΔFI defined as:

(82) $$\Delta FI=\frac{\sigma_{eq,a}-s_{-1}}{s_{-1}}=FI-1$$

is shown in Figure 9. The fatigue index error ΔFI provides a reasonable parameter showing how close the predicted equivalent stress is to a perfect prediction of the material fatigue strength in fully reversed axial loading $s_{-1}$, when ΔFI=0.0%.

From the final four items to be studied, three refer to quite brittle materials according to κ response, and only one (BKL06) to ductile material. This is the only item, which brings along a difference between results of MCC and MCE schemes for all evaluated multiaxial criteria (the output of the MCE solution gets better). For the two critical plane methods of the maximum damage type, there is no substantial change for any other test case. Results of the Matake method improve for all load cases when the scheme is switched from MCC to MCE. The output obtained for the Matake criterion for the MCE variant is very close to the output provided by the Findley method. The integral methods (PIN and LZ) show very slight changes for the brittle material, and the switch to MCE brings along some more visible improvement only for ductile material. A very important change occurs for the Crossland method. This is no wonder because this method showed an extreme dependency on the phase shift in the MCC variant (see Figure 8), and non-proportional cases resulted in too non-conservative predictions (see [21]). The MCE scheme erases any phase shift effect, which is positive here. It is quite interesting to see that the outputs of individual methods for each load cases quite substantially differ for the MCC solution, but they are getting closer to each other once the MCE concept is utilized.

The MOI concept provides a wholly different set of results, see Figure 9 bottom. All predictions for brittle materials are getting extremely conservative, while the only representative of ductile material—BKL06 test—results in the prediction better than the ones obtained for MCE or MCC concepts.

The outcome of this section is somehow limited. Only four test items were studied, which is quite a small number to observe some more general trend. Furthermore, the AMSD25 set, from which these items were derived, focuses on experiments for which the prediction results of common multiaxial methods are either substantially imprecise or where great differences are found among various estimation methods. It is of interest, therefore, if the observed behaviour is more general.

### 4.2. Out-of-Phase Experiments from FatLim Database

To find more general trends in the response to out-of-phase loading, the original FatLim set of 288 items, from which the AMSD25 data set is retrieved in [17], is evaluated. The difference between individual stress path evaluation methods is observable only for load cases, where there is some non-zero phase shift on any load channel. This means, 74 data items from the whole set can be selected to fulfil this condition, from which 29 data items are free of any mean stress influence. The statistical summary of all computations is provided in Table 2 on the 74 data items including all out-of-phase load cases in FatLim and in Table 3, which refers to 29 data items selected from the previous group by omitting all load cases with any non-zero mean stress. The differences between results for the MCC and MCE concepts in each of these two tables are similar, but the absolute values document the worse response of the Findley criterion and of the Matake criterion to the mean stress affected cases (included in Table 2). It is interesting that when hereafter the trend of changes when switching from MCC to MCE is commented, it usually gets the most visible manifestation on load cases, which are free of any mean stress.

## 5. Discussion

The various partial analyses shown previously were designed to form complementary indications on how the various multiaxial fatigue strength criteria respond to different procedures of stress path processing. This Discussion section responds mostly to the results provided in Table 2 and Table 3, but the found information corresponds well with previous findings described in Section 3 and Section 4. The MOI concept showed itself imprecise already in the previous sections, and the results in both those tables only confirm this conclusion. The results for applying the MOI concept to any multiaxial fatigue strength criterion are too conservative once the phase shift between the load channels gets non-zero. It is obvious that the improvement assumed by Meggiolaro and Castro e.g., in [44] to occur compared with MCC is not fulfilling the expectations—the magnitude of the effect of de-phasing is too big. For that reason, the differences in the output of MCE and MCC will be focused on in the discussion above all.

The PCRN criterion and the Findley criterion work with the same concept of the search for the critical plane given by the maximum damage (i.e., maximum equivalent stress) obtained. The change in trends for both of them is therefore very similar. MCE shifts some of the results to more conservative estimates compared with MCC. The analysis of data in the FatLim database shows that this change concerns the cases of extra-ductile materials (and ductile materials to a lesser scale) for which the load ratio with prevalent axial stress over shear stress was applied. This is complies with the previous observations based on Figure 3 and Figure 4. For all these cases, the output obtained while using the MCE concept is closer to good prediction (i.e., ΔFI=0.0%) than the MCC concept can provide. Because other items in the test set are insignificantly affected, the conclusion is quite obvious—the use of the MCE concept brings along positive changes compared with MCC as it provides the generally better output.

**Figure 10:**
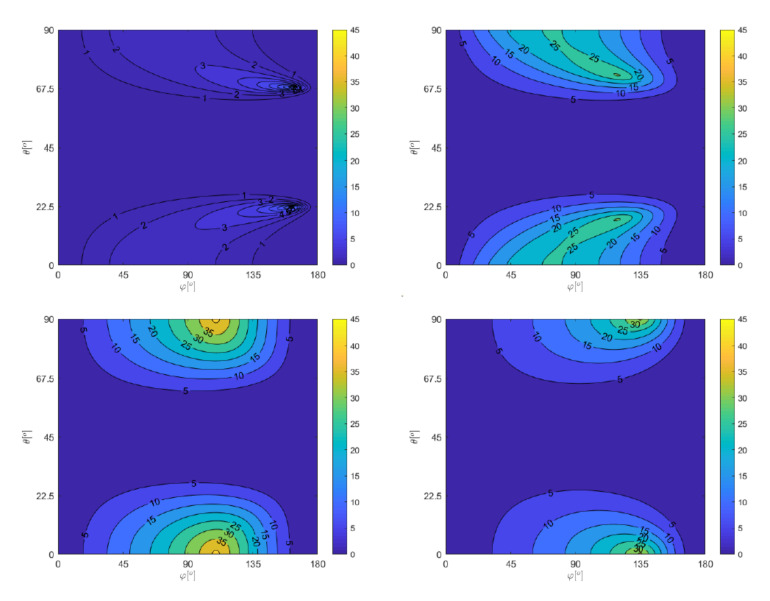
The difference between shear stress amplitudes $C_{a}$ retrieved by either the MCE concept or by the MCC concept ($C_{a,MCE}-C_{a,MCC}$) for various Euler angles φ and θ and for various stress ratios: $r_{\sigma }=0.29$ top left, $r_{\sigma }=1.00$ top right, $r_{\sigma }=1.73$ bottom left and $r_{\sigma }=2.63$ bottom right.

The explanation as to why the cases with prevalent axial stress over shear stress are more affected can be manifested very well on graphs in Figure 10. The maps of $C_{a,MCE}-C_{a,MCC}$ differences over both Euler angles φ and θ are presented there. Each of four maps represents the distribution of this difference for another stress ratio for the combination of axial loading and of torsion with the phase shift of 90 deg. For the chosen four variants or $r_{\sigma }$, the output documents that the highest difference between the MCE and MCC concepts can be expected for $r_{\sigma }=1.73$ and for higher values to a slightly lesser extent. If the stress ratio is small (i.e., shear stress is prevalent over axial stress), the differences are very small. Because the normal stress amplitude $N_{a}$ is not affected by the stress path description method, this effect is further decreased once the complete equivalent stress amplitude is computed.

There are 17 cases in the FatLim database in which the Matake criterion gets to higher ΔFI values when switching from MCC to MCE. On these items, the mean value of ΔFI=−11.1% reached by the MCC is shifted into the mean ΔFI=−3.2% if the MCE concept is used. This change is thus positive. It concerns the cases with prevalent axial stress over shear stress (mostly stress ratios above 2.00). All material types are affected. The change affects however also the other side of the ΔFI range—the use of MCE can also decrease the resulting ΔFI. For 27 data items, on which this change is observed, the mean ΔFI=12.6% obtained from MCC moves to ΔFI=4.4% of MCE. Among those cases, also load cases with more non-zero normal stresses and with various mean stresses involved can be found. The simple axial-torsion load cases can be found even here, but usually with the stress ratio between 1.73 and 2.00.

If the change related to the switch from the MCC concept to the MCE concept was observable only for some items from those OOP cases studied with the critical plane criteria above, the change by integral methods (PIN and Liu & Zenner) affects most of the evaluated load cases. This observation has a logical reason hidden again in maps presented in Figure 10. Only in the case of small stress ratio, the difference in $C_{a,MCE}$ and $C_{a,MCC}$ parameters can be assumed negligible over whole ranges of Euler angles. Once the difference is more substantial, the integration of the complete parameter over all angles must project it into the equivalent stress amplitude. Except for one brittle material and the load case with the stress ratio of 2.00, the Liu & Zenner method leads to higher ΔFI, when transiting from MCC to MCE. The most affected cases are those where materials with κ>1.6 are loaded. If 20 most affected load cases are evaluated, the shift from mean ΔFI=0.3% for MCC to ΔFI=7.6% for MCE results in the obvious conclusion that the MCE concept does not seem to be the right choice for the Liu & Zenner method. Very similar findings can be written on the evaluation of the PIN method. The shift for 20 most affected load cases shows the mean ΔFI=2.5% of MCC to be moved to ΔFI=10.7% of MCE. The load cases with higher κ (κ>1.60) are the most affected by selecting the stress path description concept. The outputs of the PIN method for both concepts also differs for the brittle material, where the PIN method gives about one percent lower ΔFI values, getting them closer to zero. Overall, the output of the MCE concept for both integral methods is inferior to the use of the MCC scheme.

**Figure 11:**
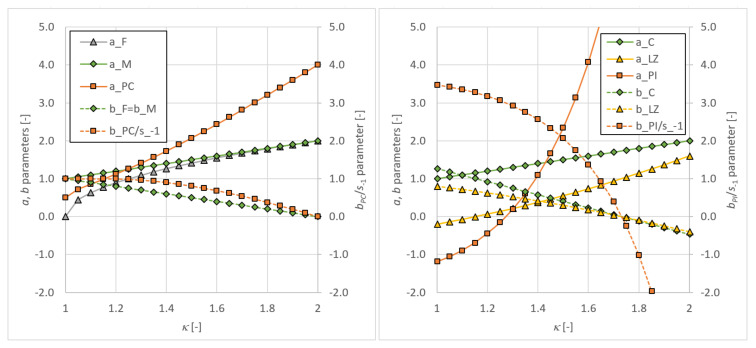
Material parameters of criteria described in Equations (70)–(75).

One point has not been discussed until now. How it could occur for some stress combination that the integral method can lead to lower FI parameters if the MCE concept replaces the MCC concept? If the stress path is elliptic, the MCE should generate higher (or at least equal) output $C_{a}$ than MCC, and thus the integration over whole ranges of both Euler angles should provide higher FI for MCE than MCC could give. In the previous paragraph, exceptions to this finding were noted. Additionally, checks in Figure 6 and Figure 7 manifest that such behavior could occur commonly for brittle materials. The explanation can be found in Figure 11. The change of the stress path description strategy affects only the shear stress amplitude $C_{a}$, while the normal stress amplitude $N_{a}$ is left untouched but this selection. While all evaluated critical plane theories result in positive *a* material parameters over the whole range of κ fatigue strength ratios, this is not true for integral methods, which for low κ typical for brittle materials result in negative values. The negative value of *a* parameter causes the $C_{a}$ shear stress amplitude, increased by the MCE concept compared to MCC application, to decrease the final amplitude of equivalent stress $\sigma_{eq,a}$.

In the case of the Crossland criterion, the change when switching from MCC to MCE is very profound. The well-visible non-conservativeness provided when MCC is used is avoided when MCE is applied. For many cases (but the most visible is the change for extra-ductile materials) the ΔFI changes by 20–35%. The Crossland method benefits from switching to MCE from MCC, as can be manifested by the output statistics available in Table 2 and Table 3.

Though the output seems quite conclusive, open questions remain. The MCE concept proved itself to be the best choice for the critical plane criteria and also for the Crossland method processed in the Ilyushin deviatoric space. The validation, however, bases this decision only on the stress path forming the ellipse. For such stress path, the identical output will be provided by various MCE approaches (see [9]), by the MPH approach or by the minimum convex hull [12]. To differentiate among all these methods, a more complicated stress path is necessary. This requirement can be either solved by using other load signals than harmonic, or by using different load frequencies on different load channels. The latter solution, however, necessitates involving some concept of damage accumulation.

## 6. Conclusions

The paper analyzed the problem of the stress path evaluation in the multiaxial fatigue strength criteria. It started with the analysis of the most complex multiaxial non-contact load case, which is the pressurized tube loaded additionally in the axial direction and in torsion. Even if all these load signals are involved with whichever phase shifts between their harmonic functions, but with identical frequencies, the ellipse will be the output stress path either on any evaluated plane or in the Ilyushin deviatoric space (in the case of the 5D deviatoric space, its description reduces into a 3D subspace). Thanks to that, most methods invented replacing the Minimum Circumscribed Circle (MCC) result for such load cases in the shear stress amplitude identical to the Minimum Circumscribed Ellipse (MCE). Parameters of this ellipse are analytically described in this paper. In addition to those two methods, the concept of Moment of Inertia (MOI) is also analyzed in this paper.

These conclusions from various analyses provided in the paper can be made:

1. The MOI concept results in a too conservative output because it exaggerates the phase shift effect too much.
2. For the tested critical plane criteria (PCRN or by Findley), which define the critical plane by the maximum damage reached, the use of MCE improves the prediction quality in comparison with using MCC.
3. For the Matake critical plane criterion using the maximum shear stress range to select the critical plane, MCE largely improves the quality of estimates compared with MCC. The use of MCE brings the prediction results close to the output of the Findley critical plane method of the maximum damage type.
4. The use of the MCE concept for the Liu and Zenner method and for the Crossland criterion results in making both methods insensitive to the phase shift effect.
5. The validated integral criteria seem to lose some of the prediction quality when using the MCE concept. The use of MCC should be preferred.
6. The Crossland method benefits from the switch from MCC and MCE, as the latter solution mends its too non-conservative output for out-of-phase loading discussed e.g., in [21]. This change makes the criterion insensitive to the phase shift.

## Figures and Tables

**Table 1:** Data items retrieved from AMSD25 test set by selecting only the load cases without the mean stress effect and with the non-zero phase shift.

ID	Material	$\sigma_{x,a}$ [MPa]	$\tau_{\mathrm{xt},a}$ [MPa]	$\delta_{\tau }$ [deg]	$s_{-1}$ [MPa]	$t_{-1}$ [MPa]	κ [-]	$r_{\sigma }$ [-]
AR1004	42CrMo4	481.6	278.1	90	488.2	403.8	1.21	1.73
BKL06	100Cr6	607.0	303.5	90	866.0	541.0	1.60	2.00
HeG02	GGG-60	221.0	110.5	90	275.0	249.0	1.10	2.00
PSSB003	X2CrNiMo17-12-2	302.0	151.0	90	313.0	258.6	1.21	2.00

**Table 2:** Results of the multiaxial fatigue strength estimations compared by the statistical evaluation of the ΔFI output and for different concepts of processing the load path. All out-of-phase load cases from the FatLim database used.

ΔFI	PCN	PCN	PCN	FIN	FIN	FIN	MAT	MAT	MAT
Statistics	MCC	MCE	MOI	MCC	MCE	MOI	MCC	MCE	MOI
average	−0.2%	1.1%	12.3%	7.8%	9.3%	23.2%	8.5%	7.3%	21.7%
st.dev.	6.4%	6.0%	8.9%	17.7%	16.6%	16.9%	20.0%	17.3%	14.7%
max.	16.3%	16.3%	33.1%	41.8%	41.8%	62.4%	46.9%	46.9%	61.1%
min.	−18.0%	−18.0%	−10.8%	−39.8%	−39.8%	−23.7%	−51.3%	−51.3%	−18.4%
ΔFI	**PIN**	**PIN**	**PIN**	**L&Z**	**L&Z**	**L&Z**	**CROSS**	**CROSS**	**CROSS**
**Statistics**	**MCC**	**MCE**	**MOI**	**MCC**	**MCE**	**MOI**	**MCC**	**MCE**	**MOI**
average	2.9%	6.0%	14.4%	−2.5%	1.1%	13.6%	−12.3%	2.2%	19.0%
st.dev.	7.0%	7.9%	10.0%	11.2%	12.4%	16.6%	13.2%	12.1%	16.5%
max.	22.8%	25.4%	37.1%	23.0%	27.5%	52.2%	17.9%	29.8%	55.8%
min.	−10.0%	−9.8%	−9.8%	−54.6%	−54.6%	−54.6%	−47.4%	−33.0%	−19.5%

**Table 3:** Results of the multiaxial fatigue strength estimations compared by the statistical evaluation of the ΔFI output and for different concepts of processing the load path. Out-of-phase load cases from the FatLim database used, while excluding cases with non-zero mean stresses.

ΔFI	PCN	PCN	PCN	FIN	FIN	FIN	MAT	MAT	MAT
Statistics	MCC	MCE	MOI	MCC	MCE	MOI	MCC	MCE	MOI
average	−0.7%	2.0%	17.2%	−0.4%	3.1%	23.1%	2.2%	4.1%	23.0%
st.dev.	7.1%	6.2%	8.2%	10.9%	9.4%	13.1%	15.6%	9.7%	12.6%
max.	16.3%	16.3%	33.1%	30.1%	30.1%	62.4%	46.9%	31.5%	61.1%
min.	−11.7%	−9.7%	3.7%	−17.7%	−12.6%	4.5%	−19.6%	−12.5%	3.3%
ΔFI	**PIN**	**PIN**	**PIN**	**L&Z**	**L&Z**	**L&Z**	**CROSS**	**CROSS**	**CROSS**
**Statistics**	**MCC**	**MCE**	**MOI**	**MCC**	**MCE**	**MOI**	**MCC**	**MCE**	**MOI**
average	0.8%	5.9%	16.3%	−1.2%	3.8%	17.5%	−14.3%	5.4%	26.6%
st.dev.	5.5%	8.2%	9.7%	8.4%	9.9%	11.3%	8.6%	9.3%	12.5%
max.	14.2%	21.9%	35.7%	19.4%	20.3%	50.0%	3.2%	27.2%	55.8%
min.	−10.0%	−6.5%	1.2%	−15.7%	−12.8%	−0.3%	−30.9%	−10.8%	5.2%

## Data Availability

Data sharing not applicable

## Abbreviations

The following abbreviations are used in this manuscript:

AMSD25	dataset defined in [17]
CROSS	Crossland criterion
FIN	Findley criterion
IP	in-phase loading
LZ	Liu and Zenner criterion
MAT	Matake criterion
MCC	Minimum Circumscribed Circle
MCE	Minimum Circumsribed Ellipse
MOI	Moment of Inertia
MPH	Maximum Prismatic Hull
OOP	out-of-phase loading
PCRN	Papuga critical plane criterion in the newer version, see [19]
PIN	Papuga integral criterion in the newer version, see [23]

## Nomenclature

*a*,*b*,*c*,*d*	[-]	material parameters of various multiaxial fatigue strength estimation methods
*C*	[MPa]	shear stress on an examined plane
δ	[deg]	phase shift of the given stress signal compared with the axial stress signal
ΔFI	[%]	fatigue index error
FI	[-]	fatigue index
$\sqrt{J_{2}}$	[-]	second invariant of the stress tensor deviator
κ	[-]	ratio of fatigue strengths in fully reversed loadings ($s_{-1}/t_{-1}$)
*N*	[MPa]	normal stress on an examined plane
$r_{\sigma }$	[-]	stress ratio ($\sigma_{x}/\tau_{xt}$)
*s*	[MPa]	fatigue strength in axial loading
σ	[MPa]	nominal axial stress induced by axial load
$\sigma_{H}$	[MPa]	hydrostatic stress
*t*	[MPa]	fatigue strength in torsion
τ	[MPa]	nominal shear stress induced by torsion
θ, φ	[deg]	Euler angles defining the orientation of the examined plane

## Indexes

0	related to repeated loading (from 0 to val, where val is the referred value)
−1	related to fully reversed loading (from −val to val, where val is the referred value)
*a*	amplitude
eq	equivalent
*m*	mean value
max	maximum value
*r*	radial direction
*t*	tangential direction
*x*	axial direction
